# First Case of Dual Size Asymmetry in an Identical Arthropod Organ: Different Asymmetries of the Combative (Sexual) and Cutting (Non-Sexual) Parts of Mandibles in the Horned Stored-Product Beetle *Gnatocerus cornutus* (Fabricius, 1798)

**DOI:** 10.3390/insects9040151

**Published:** 2018-10-29

**Authors:** Tomas Vendl, Vaclav Stejskal, Radek Aulicky

**Affiliations:** Crop Research Institute, Drnovska 507/73, Prague 6-Ruzyne CZ-16106, Czech Republic; stejskal@vurv.cz (V.S.); aulicky@vurv.cz (R.A.)

**Keywords:** asymmetry, mandibles, secondary sexual trait, sexual dimorphism, stored-product pest

## Abstract

Although it is known that separate insect body structures may be asymmetrical within one species, the different functional asymmetries within a single organ as a result of differential selective regimes have not been described. Based on microscopic measurements and SEM photography, we examined the size, shape and asymmetry of the mandibular structures of males and females of the sexually dimorphic broad-horned flour beetle, *Gnatocerus cornutus* (Tenebrionidae, Coleoptera). It was found that sexual dimorphism only manifests in certain outgrowth parts (horns) of male mandibles, while the remaining cutting parts of the mandibles hold identical morphologies for both sexes. A more interesting finding—since this is the first published case of dual functionally selected asymmetry in an identical arthropod organ—was that the cutting part of the male mandible exhibited directional asymmetry, whereas the outgrowth horn part of the mandible showed a high degree of symmetry. Moreover, there was no relationship between the size and asymmetry of horns. The results indicate different regulatory mechanisms of sexually selected combative horns and the food-functional, more conservative (constrained by hard food and adult long life) cutting parts of mandibles.

## 1. Introduction

**Figure 1:**
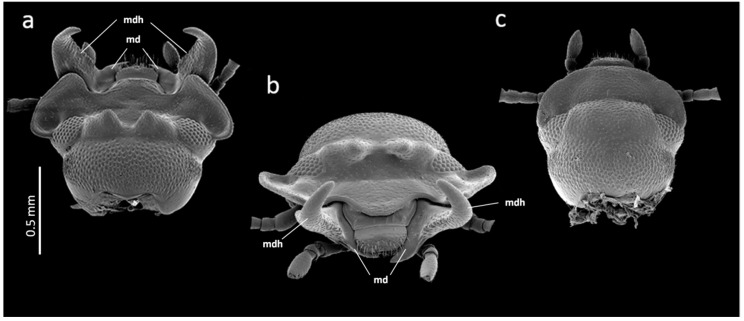
SEM micrographs of the head of *Gnatocerus cornutus*. (**a**) Dorsal view of a male head, (**b**) frontal view of a male head, (**c**) dorsal view of a female head. md: mandible; mdh: mandibular horn.

The broad-horned flour beetle, *Gnatocerus cornutus*, is a tenebrionid stored product pest that is currently on a geographical spread (e.g., it was recently discovered in Japan in 2016 [1]). The species is often found in flour mills, grain warehouses and other facilities [2], and it is morphologically and physiologically adapted to feed on and digest more than 60 various stored agricultural commodities [3]. Not only larvae but also adults are pestilential since they can live and feed upon human resources for up to several months. Apart from the species’ importance in agriculture and the food industry, it is interesting to study from the perspective of behavioral and morphological studies. It is the only synanthropic stored product pest that shows social behavior, and males have fully developed functional combative mandibular horns on their mouthparts (Figure 1a,b). Since horns are used as weapons in male–male fights [4], *G. cornutus* has recently become a model organism in the insect ethology and evolution of sexual traits (e.g., [4,5,6]). As mentioned above, the *G. cornutus* adult is a polyphagous and long-lived, and therefore, its mandibles must be functionally adapted not only for fighting (in contrast to, e.g., male stag beetles) but also for physically processing food (e.g., cereals) of varied hardiness. The two different functional roles of one organ thus potentially constitute a striking interplay and trade-off between selective regimes in the development, size and symmetry of the two different parts of the organ.

Although the general body plan is bilaterally symmetrical in the majority of animals, most paired organs manifest more or less pronounced degrees of asymmetry. There can be asymmetry in size as well as in shape; the latter being a widespread phenomenon in the animal kingdom [7]. The most common form of asymmetry is a fluctuating asymmetry (FA). FA is defined as a random deviation from perfect symmetry in paired traits, with no tendency for one side to be larger than the other at the population level [8,9,10]. FA originates as a consequence of an individual’s inability to deal with different kinds of environmental stresses [8,9]. Directional asymmetry (DA) is another type of asymmetry, which occurs when all individuals have an enlarged left or right side. This type of asymmetry is usually adaptive. The mandibles of some insects represent an example of DA. Notable mandible DA is reported in snail-feeding species [11,12], but different morphologies of the left and right mandible are common in insects [13,14,15]. Apparently, such asymmetry has a functional aspect, as it allows the mandibles to fit into each other and thus helps to process food.

Secondary sexual traits (i.e., weapons and ornaments) represent, from a symmetry perspective, a rather special case. It is generally believed that sexual selection favours symmetry [16,17], and, indeed, excluding a few exceptions such as chelae of Crustacea [18], sexual traits exhibit symmetry as a general pattern in the majority of groups [19]. Moreover, because sexual traits are thought to be costly to produce, some studies suggest that their FA is a good indicator of male quality [16,20]. Nevertheless, a relatively large amount of studies failed to find a negative relationship between weapon size and FA [21,22,23], and so there is controversy about FA as an indicator of males’ quality.

Horns, enlarged mandibles, prolonged forelimbs and other exaggerated structures have developed in a variety of beetle lineages [24]. Typically, they are present only in males [25] and serve as weapons in male–male combats [26]. Their presence is often accompanied by sexual dimorphism in size [27]. The weapons generally scale positively with body size: larger males have disproportionally larger traits [28], which is also the case for the horns of *G. cornutus* [4]. In some cases, there is a developmental threshold involved, whereby only males above a certain body size express the trait [28].

Mandibles in the males of *G. cornutus* play two functional roles (one in feeding and one in male–male combat), and they are thus presumably under dual selective pressure. During battles, males use de novo developed structures (horns) on their mandibles [4]. The size of these mandibular horns determines the outcome of the fight: the male with larger horns is a better competitor [4], and so horn size is subject to sexual selection. Thus, we tested whether the mandibles of both sexes exhibit DA and if the males’ horns exhibit FA. In addition, we examined if there is a negative relationship between the horn size and the predicted FA. Finally, we sought to discover whether there are changes in the mandible size, structure or function attributable to the presence of horns; i.e., whether there are any differences between males and females in mandible morphology and asymmetry.

## 2. Materials and Methods

**Figure 2:**
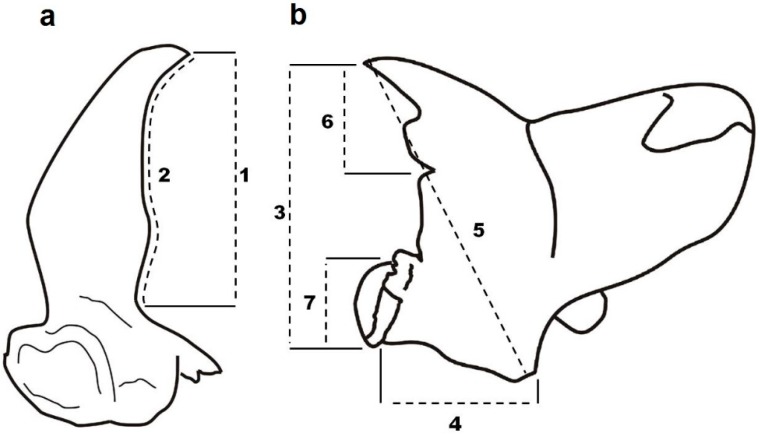
Measurements of (**a**) horns and (**b**) mandible parts of *Gnatocerus cornutus*. 1 and 2: horn length; 3: mandible length; 4: mandible width; 5: diagonal; 6: incisor length; 7: molar length.

Beetles used in the study were from a laboratory culture reared in darkness at 25 °C and 75% RH on a mixture of wheat kernel, yeast and oat flakes as food. Adults of a maximum of 4 weeks of age were used for the study to ensure low mandible wear. Beetles were dissected, and the mandibles were mounted with soluble glue on a microscope slide. The dorsal side was analyzed, and the mandibles were mounted maximally horizontally. The following measurements were made: mandible length, mandible width, diagonal, incisor length and molar length (Figure 2b). For measurements of the mandibular horns (further referred to only as horns), the positions of the males’ mandibles were subsequently changed so that the horns would be positioned horizontally (so the tip of the mandible pointed obliquely down, Figure 2a). Because the horns of *G. cornutus* are curved and because of the sensitivity of the sexual traits to measurement error [23], we conducted two measurements of each horn: (1) the length of the horn as a line from the tip to the base and (2) the border of the same distance to consider the horn curvature (Figure 2a). The measurements were made using photos taken with a stereomicroscope Olympus SZX10 equipped with a Canon 1300D digital camera and were analyzed by QuickPHOTO INDUSTRIAL 3.1 software. Three males (belonging to 21.6, 51.3 and 91.8 percentiles of body length) had extremely asymmetrical horns, as one of the horns was much more curved than the other. Because such asymmetry occurred only in a small fraction of beetles, we regarded it as a developmental defect, and the beetles were omitted from analyses dealing with horn asymmetry. Nevertheless, we performed the analyses also with the three excluded data points and the results remained almost unchanged (asymmetry of horn length 1: mean = −1.39, t_37_ = −0.37, n.s., K-S test: 0.18, n.s.; asymmetry of horn length 2: mean = 3.61, t_37_ = 3.60, n.s., K-S test: 0.08, n.s.). We also measured body length (pronotum plus elytra length). A total of 38 males and 26 females were analyzed.

All asymmetries were calculated as the right minus left side. FA is characterized by the asymmetry mean around zero with normal distribution, while DA has a normal distribution of right minus left differences whose means significantly differ from zero [9]. Thus, the type of asymmetry was determined by a one-sample t-test (whether the value differed from zero) along with a Kolmogorov–Smirnov goodness-of-fit test. The relationship between the horn and body size has been elucidated in previous studies [4,29]. To determine the relationship between the characteristic size and asymmetry, we used a Pearson’s correlation coefficient analysis. Because there are differences in the body shape depending on the body size in *G. cornutus* [30], we used body length as a measure of body size to compare the mandible size between males and females. The comparison was performed using an analysis of covariance, with the body length as a covariate. Analyses other than to ascertain the type of asymmetry were conducted on log-transformed data.

## 3. Results

### 3.1. Mandible Asymmetry

**Figure 3:**
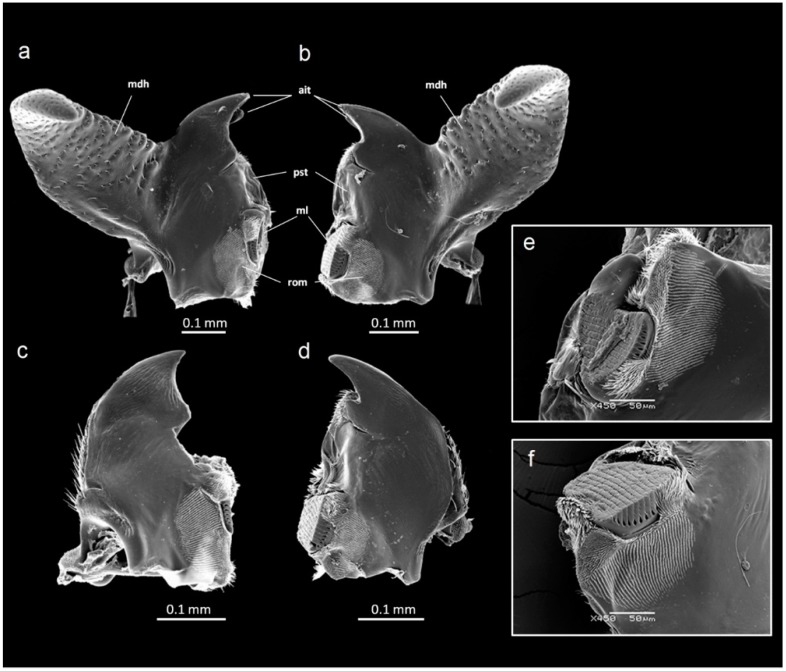
SEM micrographs of the mandibles of *Gnatocerus cornutus*. (**a**) Dorsal view of a left male mandible, (**b**) dorsal view of a right male mandible, (**c**) dorsal view of a left female mandible, (**d**) dorsal view of a right female mandible, (**e**) detail of the molar region of a left male mandible, (**f**) detail of the molar region of a right male mandible. ait: apical incisor teeth; mdh: mandibular horn; ml: mola; pst: prostheca; rom: rows of microtrichia.

In both the males and females, there was DA in mandible size (Table 1). The left mandible was always longer than the right mandible, but the opposite was true for the mandible width. Thus, the left mandible of *G. cornutus* is relatively longer and more slender than the right mandible. In addition to the mandible length and width, the mandibles in both sexes differed in other measurements, indicating that there is an additional right–left asymmetry in both mandible size and shape (Figure 3a–d): the left mandible had a longer incisive part and a longer diagonal, while the mola was longer in the right mandible. However, this dimorphism in molar size reflects differences in shape, as was revealed by the SEM (Figure 3e,f).

### 3.2. Horn Asymmetry

In contrast to the mandibles, the mean values of the horn asymmetry did not significantly differ from 0 in either of the two measures (Table 1), which indicates FA in the horn length.

### 3.3. Sexual Differences

ANCOVA with body size as a covariate revealed that the males had a relatively larger mandible length (right: F_1,61_ = 83.72; left: F_1,61_ = 78.85), mandible width (right: F_1,61_ = 263.83; left: F_1,61_ = 93.36), incisor length (right: F_1,61_ = 48.58; left: F_1,61_ = 33.81) and diagonal (right: F_1,61_ = 157.09; left: F_1,61_ = 171.03; in all cases *p* < 0.001) but not molar size (right: F_1,61_ = 0.15, *p* = 0.70; left: F_1,61_ = 0.46, *p* = 0.50). Nevertheless, the general appearance, morphological details, and pattern of asymmetry (Table 1) were similar in the males and females. There were no differences in the absolute asymmetry of mandible length (t_62_ = 1.99, *p* = 0.051) and width (t_62_ = −1.84, *p* = 0.07) between males and females.

### 3.4. Relationship of Mandible and Horn Asymmetry

There was no relationship between the horn asymmetry and the mean horn length (*r* = −0.054, *p* = 0.76), and the same was true for the mandible length (males: *r* = −0.20, *p* = 0.23, females: *r* = −0.07, *p* = 0.75) and width (males: *r* = −0.07, *p* = 0.69; females: *r* = −0.30, *p* = 0.14). Moreover, although the mean mandible and the mean horn length were highly correlated (*r* = 0.86, *p* < 0.001), the asymmetries of the mandible and the horn length were not (*r* = −0.084, *p* = 0.63).

## 4. Discussion

The relationship between mandible morphology and diet has been recognized in a variety of beetle taxa [31,32,33,34]. The functional asymmetry of mandibles is a relatively common phenomenon [13,14,15]. Apparently, such asymmetry has a functional role in the processing of food. We found that the left and right mandible of *G. cornutus* differ in shape and exhibit DA in several measurements: the left is longer but more slender, with a longer incisive part and with the mola located relatively higher (Figure 3a–d); moreover, the shape of the mola itself is dimorphic (Figure 3e,f). The mandibles resemble typical tenebrionid mandibles [35], including those of another polyphagous stored product species, *Alphitobius diaperinus* [36]. They bear standard structures used for the removal, cutting and grinding of food, such as a pair of incisor teeth, hyaline prostheca, striate mola and parallel rows of microtrichia on the dorsal molar surface. All these structures indicate a general feeding habit of the species [36] and explain its wide range of food.

Because males of *G. cornutus* possess large horns on their mandibles that serve as weapons in males’ fights [4], we initially intended to examine whether there are any associated changes in the morphology and function of male mandibles (i.e., if they can cause the same damage on stored products as those of females). Nevertheless, because there are no sexual differences in the mandible morphology nor in the form and degree of asymmetry, we concluded that the males feed in the same manner as the females. On the other hand, the males’ mandibles are, in general, larger, which is probably caused by the presence of the horns. The presence of horns on mandibles is not unique to *G. cornutus*. Similar structures also are present in the geotrupine genus, *Lethrus* [37], in *Agathidium* beetles from the family Leiodidae [38] and in another tenebrionid genus, *Molion* [39]. Also, in these cases, the horns are involved in males’ fights, and they probably developed as a consequence of a need to maintain the feeding function (in contrast to, for example, stag beetles, in which the mandibles are released from their primary function, and the mandibles per se can serve as weapons [24]).

In contrast to mandibles, we found that the horns exhibit random deviations from symmetry (i.e., FA). This finding is consistent with other studies that suggest that sexual selection favours symmetry (review in [17]). Large males of the stag beetle *Prosopocoilus inclinatus* have symmetrical mandibles, while the mandibles of females are asymmetrical [40]. The authors suggest that symmetrical mandibles of males are favored in male–male combats, while mandibles of females are using for the cutting of wood fibers. A similar situation may occur also in *G. cornutus* (the symmetrical parts of mandibles are advantageous in combats, and the asymmetrical parts are adapted for the processing of food). There are presumably different selective regimes between sexual and nonsexual morphological traits, because sexual traits occur with strong directional selection in contrast to other nonsexual traits, which are rather subject to stabilizing selection [16]. The presence of two forms of asymmetry in one organ (i.e., horns and the mandibles per se) can be particularly noteworthy from a developmental perspective. There is often poor correlation between the asymmetries of different body parts [9], but this could easily be a consequence of different timings of development. We assume that this is not the case for the mandibles and horns (i.e., two parts of a single organ) of *G. cornutus*. This is further supported by the fact that, although there is a strong correlation between the horn and mandible size, there is no correlation between their asymmetries.

This work represents another study which fails to find a negative correlation between the size and asymmetry of a secondary sexual trait, and it thus does not support the claim that the level of FA is an indicator of male quality. One of the assumptions behind the theory that FA signals male quality is that the secondary sexual traits are costly to produce [16], and thus, only superior males are able to produce highly developed and symmetrical sexual traits. In *G. cornutus,* the mandibular horns are costly in terms of decreased pupal survival [41] and a reduction of wing and elytra size [42]. Nevertheless, the females determine whether copulation occurs [29] and, likely because daughters sired by large males have a lower fitness (because of their suboptimal body shape) and because of harassment by the superior males [43], males with large horns are not preferred by females [29]. It is questionable how counteractive female choice is to horn size in *G. cornutus*, but it is possible that the horns are not at absolute limit of production.

## 5. Conclusions

As far as we know, this study provides the first case of different forms of size asymmetry of the sexual and non-sexual parts of a single arthropod organ. In accordance with the hypothesis, we proved that males’ mandibular horns exhibit FA, while mandibles per se of both sexes are directionally asymmetric (DA). Nevertheless, further research is needed to elucidate whether the horn symmetry is under sexual selection, i.e., whether symmetric males are favored in male–male combats or in female choice. Finally, we believe that further insights into the issue may provide research on other suitable organisms, e.g., the genus *Lethrus* with more diverse mandibular horns.

## Figures and Tables

**Table 1:** Differences (in μm) between the right and left sides of several mandible parts of *G. cornutus* males and females, and male horn lengths 1 and 2. Statistics: one sample *t*-test and Kolmogorov–Smirnov test.

Trait	Mean Asymmetry ± SE	*t* (df)	K-S Test	Larger Side	Asymmetry Pattern
Males					
Mandible length	‒23.97 ± 1.70	‒14.12 (37) ***	0.11 (n.s.)	L	DA
Mandible width	22.05 ± 1.53	14.42 (37) ***	0.13 (n.s.)	R	DA
Diagonal	‒11.55 ± 1.57	‒7.37 (37) ***	0.15 (n.s.)	L	DA
Incisor length	‒18.13 ± 1.22	‒19.92 (37) ***	0.11 (n.s.)	L	DA
Molar length	4.18 ± 1.27	3.29 (37) **	0.11 (n.s.)	R	DA
Horn length 1	1.17 ± 2.09	0.56 (34)	0.07 (n.s.)	-	FA
Horn length 2	4.46 ± 2.47	1.81 (34)	0.11 (n.s.)	-	FA
Females					
Mandible length	‒18.69 ± 2.03	‒9.19 (25) ***	0.15 (n.s.)	L	DA
Mandible width	18.08 ± 1.35	13.39 (25) ***	0.15 (n.s.)	R	DA
Diagonal	‒13.58 ± 2.29	‒11.41 (25) ***	0.15 (n.s.)	L	DA
Incisor length	‒19.35 ± 1.70	‒5.94 (25) ***	0.14 (n.s.)	L	DA
Molar length	4.58 ± 1.68	2.73 (25) *	0.18 (n.s.)	R	DA

*** *p* ≤ 0.001, ** *p* ≤ 0.01, * *p* ≤ 0.05.
